# High Body Mass Index is Associated with Elevated Blood Levels of Progerin mRNA

**DOI:** 10.3390/ijms20081976

**Published:** 2019-04-23

**Authors:** Moritz Messner, Santhosh Kumar Ghadge, Thomas Schuetz, Herbert Seiringer, Gerhard Pölzl, Marc-Michael Zaruba

**Affiliations:** Department of Internal Medicine III, Cardiology and Angiology, Medical University Innsbruck, 6020 Innsbruck, Austria; moritz.messner@i-med.ac.at (M.M.); santhosh.ghadge@i-med.ac.at (S.K.G.); thomas.schuetz@i-med.ac.at (T.S.); herbert.seiringer@student.i-med.ac.at (H.S.); gerhard.poelzl@i-med.ac.at (G.P.)

**Keywords:** aging, obesity, BMI, inflammation, lamin A/C, progerin, longevity

## Abstract

Obesity is a well-described risk factor resulting in premature aging of the cardiovascular system ultimately limiting longevity. Premature cardiac death and aging is the hallmark of Hutchinson–Gilford syndrome (HGPS), a disease caused by defined mutations in the lamin A gene leading to a shortened prelamin A protein known as progerin. Since small amounts of progerin are expressed in healthy individuals we aimed to investigate the association of Body-Mass-Index (BMI) with respect to expression of progerin mRNA in blood samples of patient with known cardiovascular disease. In this cross-sectional retrospective analysis, 111 patients were consecutively included of which 46 were normal (BMI < 25 kg/m^2^) and 65 overweight (BMI ≥ 25.0 kg/m^2^). Blood samples were analyzed for quantitative expression of progerin mRNA. Progerin as well as high-sensitive C-Reactive Protein (hs-CRP) levels were significantly upregulated in the overweight group. Linear regression analyses showed a significant positive correlation of BMI and progerin mRNA (*n* = 111; *r* = 0.265, *p* = 0.005), as well as for hs-CRP (*n* = 110; *r* = 0.300, *p* = 0.001) and for Hb1Ac (*n* = 110; *r* = 0.336, *p* = 0.0003). Our data suggest that BMI strongly correlates with progerin mRNA expression and inflammation. Progerin might contribute to well described accelerated biologic aging in obese individuals.

## 1. Introduction

Excess weight is growing in frequency globally, affecting all ethnicities and genders [1] and is based on multifactorial reasons as environmental and socio-economic conditions, imbalance between caloric intake and energy expenditure [2,3], as well as genetic factors. Obesity is associated with morbidity and premature mortality and represents a major risk factor for many diseases especially cardiovascular disease [4]. It is linked to a significant decrease in life expectancy of 5–10 years in comparison to persons with Body-Mass-Index (BMI) between 22.5 to 24.9 [5]. An elevated BMI, adipose tissue [6,7] and muscular fat depositions, respectively, have been associated with aging [8].

Aging is defined as deterioration of cellular and organ function with time [9] related to many physiologic and phenotypical changes and represents the strongest risk factor for myocardial infarction, stroke, diabetes, and cancer [10].

Therefore, premature aging-like syndromes such as Hutchinson–Gilford progeria syndrome (HGPS) are of particular interest in exploring pathophysiological changes of aging processes related to cardiovascular disease.

HGPS is based on mutations influencing the precise encoding and processing of lamin A (LMNA) an important filament protein in the nucleus of eukaryotic cells [11]. LMNA is involved in the correct forming of a filamentous meshwork between chromatin and the nuclear membrane, keeping the nuclear envelope upright [12], which is essential to regulate processes like DNA replication, DNA repair and RNA transcription [13,14].

Individuals suffering from HGPS exhibit early cardiovascular atherosclerosis and often die due to heart attack and stroke as teenagers [15]. Toward the end of life, HGPS patients also suffer from heart failure due to cardiac fibrosis and cardiomegaly [16].

In most HGPS cases, a single point mutation (LMNA 1824 C > T, G608G) activates a cryptic splicing site causing the production of 50 amino acids truncated prelamin A called progerin. Progerin lacks the cleavage site for zinc-metalloproteinase (ZMPSTE24) resulting in accumulation in the nucleus, leading to disturbed lamina, telomere and DNA damages, apoptosis, early cellular senescence, and finally to deterioration of organ function [9,17].

Astonishingly, Scaffidi et al. have shown that low amounts of progerin mRNA derived by alternative splicing are also expressed in healthy individuals leading to the discussion of the role of progerin in normal aging by various groups [18,19].

Since obesity and premature aging are both accompanied with an increased cardiovascular morbidity and mortality [20], we aimed to investigate the association of BMI with respect to progerin mRNA expression in the blood of individuals with known cardiovascular disease presenting in an outpatient cardiology clinic.

## 2. Results

### 2.1. Progerin mRNA is Detectable in Human Blood Samples

**Figure 1:**
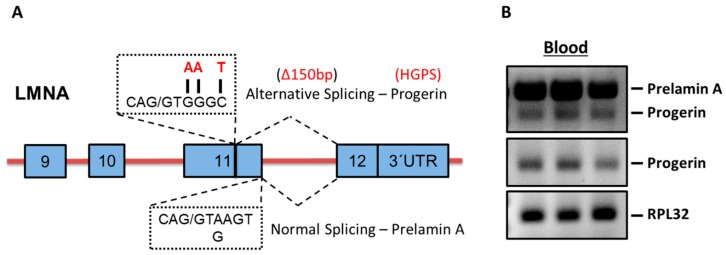
Detection of progerin mRNA expression in human blood cells. (**A**). Schematic diagram showing the cryptic splicing site as well as the three most common mutations (letters in red) in exon 11 leading to alternative splicing and progerin mRNA expression. Below the consensus donor splice sequences at the end of exon 11 for normal splicing of LMNA is depicted. (**B**). RT-PCR (Polymerase Chain Reaction) analysis from three human blood samples utilizing set of primers spanning exon 9 and exon 12 detecting both prelamin A and progerin (upper box), primers specifically detecting progerin (exon 11 and exon 12, middle box) and RPL32 as a housekeeping gene (lower box).

Figure 1A depicts the schematic Real Time-Polymerase Chain Reaction (RT-PCR) based strategy to analyze progerin mRNA expression in human blood samples. Shown are the cryptic splice site as well as the three most common mutations (>90%, sequences in red) in HGPS patients in exon 11 leading to alternative splicing and progerin mRNA expression. Below the consensus donor splice sequences at the end of exon 11 for normal splicing of LMNA is depicted.

**Figure A1:**
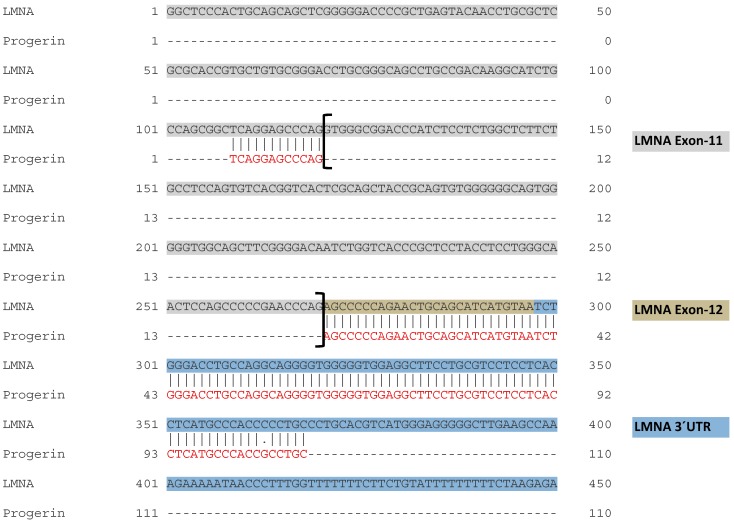
Sequencing of progerin PCR product cDNA confirms alternative splicing in the blood. Sequence alignment of purified progerin PCR cDNA (red letters) with the genomic sequence of LMNA between exon 11 (grey) to exon 12 (green) with the 3´UTR sequence (blue) revealed the anticipated gap of 150bp between exon 11 to exon 12 confirming alternative splicing of progerin.

Figure 1B shows representative RT-PCR gel bands derived from three human blood samples utilizing a set of primers spanning exon 9 and exon 12 of the LMNA gene detecting both prelamin A and the alternative splicing product progerin (upper box). In the middle box, the specific expression of the alternative splicing product progerin utilizing primers spanning exon 11 and exon 12 is shown. Ribosomal protein L32 (RPL32) was used as housekeeping gene (lower box). Further sequencing of the purified specific progerin PCR product revealed the anticipated gene sequence with a gap of 150 bp between exon 11 and 12 confirming alternative splicing of progerin in the blood (Figure A1).

### 2.2. Characteristics of Study Groups

Of the total 111 patients consecutively included, 46 (41%) revealed a BMI < 25 kg/m^2^ and 65 (58%) showed a BMI ≥ 25 kg/m^2^. Baseline clinical characteristics of the entire study population and patient groups with BMI < 25 kg/m^2^ and BMI ≥ 25 kg/m^2^ are depicted in Table 1.

Most of the patients suffered from dilatative cardiomyopathy (DCMP), followed by ischemic cardiomyopathy (ICMP), hypertrophic cardiomyopathy, and other cardiovascular disease. There were no statistical differences in the distribution of diseases between the groups (Table A1).

Renal function (GFR, creatinine) and liver parameters (γ-GT, GOT) were well balanced between the groups with no significant differences. Moreover, characteristics of over- and normal-weight individuals did not significantly differ in age, left ventricular function (LVEF) and natriuretic-peptide.

Higher BMI was significantly associated with worsening of dyspnea on exertion expressed as a higher NYHA (New York Heart Association) functional class. There were significant differences between the two groups for high-sensitive C-reactive protein (hs-CRP) (*p* = 0.01), hemoglobin A1c (HbA1c) (*p* = 0.017), triglycerides (*p* = 0.04), and high-density-lipoprotein (*p* = 0.003).

### 2.3. Progerin mRNA and CRP Were Upregulated in the Overweight Group

**Figure 2:**
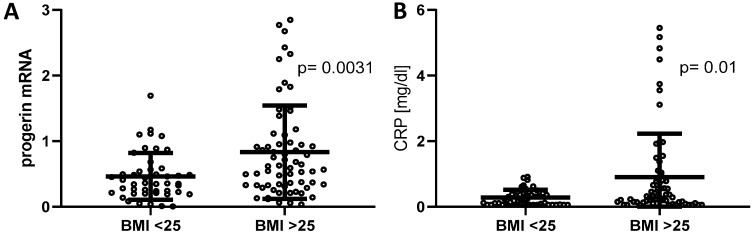
High Body-Mass-Index (BMI) is related to increased progerin mRNA and systemic inflammation. (**A**) Scatterplot diagram showing all individual data points; lines show mean values ± SD of progerin mRNA related to the reference gene RPL32 in normal (*n* = 46) and overweight individuals (*n* = 65) (*p* < 0.003 between the weight groups). (**B**) Scatterplot diagram showing all individual data points; lines show mean values ± SD of hs-CRP RPL32 in normal (*n* = 46) and overweight individuals (*n* = 64) (*p* < 0.01 between the weight groups).

As shown in Figure 2A progerin mRNA levels were significantly upregulated in blood samples of individuals with BMI ≥ 25 kg/m^2^ (*n* = 65; 0.46 ± 0.16 vs. 0.83 ± 0.71, *p* = 0.003) compared to the normal weight group (*n* = 46). Moreover, patients with BMI ≥ 25 kg/m^2^ (*n* = 64) exhibited significantly higher CRP values (Figure 2B) as the normal weight group (*n* = 46; 0.28 ± 0.24 vs. 0.9 ± 1.33, *p* = 0.01), indicating a higher activity of systemic inflammation (*p* = 0.01). Multivariate logistic regression analyses revealed CRP (OR, 3.58 (95% confidence interval, 1.19–10.76); *p* = 0.02) and progerin mRNA (OR, 3.47 (95% confidence interval, 1.31–9.19); *p* = 0.012) as independent variables.

### 2.4. BMI Correlates Positively with Progerin mRNA, CRP, and HbA1c

We further performed linear regression analyses of the total population (*n* = 111) based on Spearman’s correlation to calculate whether BMI correlates to progerin, CRP (*n* = 110) and HbA1c (*n* = 110).

**Figure 3:**
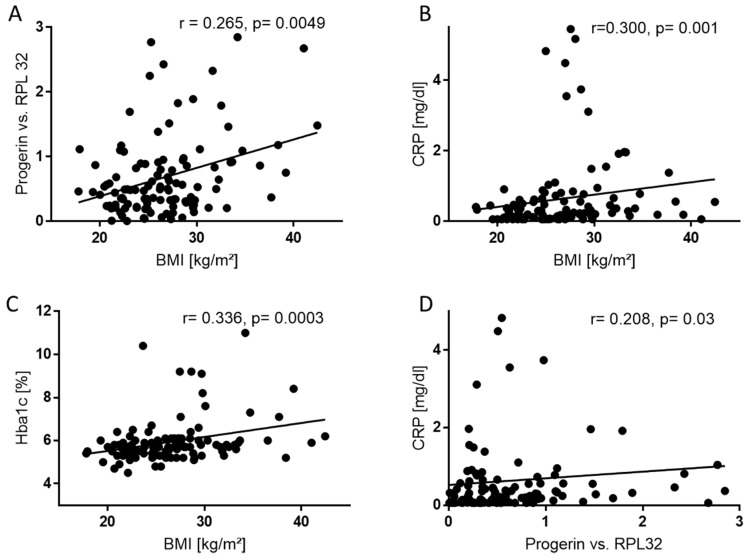
Progerin mRNA positively correlates to BMI and CRP. (**A**) Scatter plot showing the positive correlation (*r* = 0.265) between the relative amount of progerin mRNA related to RPL32 with BMI in human blood samples (*n* = 111). ** *p* ≤ 0.01. (**B**) Scatter plot showing the positive correlation (*r* = 0.300) between hs-CRP related to BMI in human blood samples (*n* = 110). ** *p* ≤ 0.01. (**C**) Scatter plot showing the positive correlation (*r* = 0.336) between Hb1Ac related to BMI in human blood samples (*n* = 109). *** *p* ≤ 0.001. (**D**) Scatter plot showing the positive correlation (*r* = 0.300) between hs-CRP related to progerin mRNA in human blood samples (*n* = 110). * *p* ≤ 0.05.

Consistent with the findings above, linear regression analysis showed a positive correlation of BMI and progerin mRNA (Figure 3A) levels in patients’ blood (*r* = 0.265, *p* = 0.005). BMI and CRP (Figure 3B) (*r* = 0.300, *p* = 0.001) as well as BMI and HbA1c (Figure 3C) (*r* = 0.336, *p* = 0.0003) were also significantly positively correlated. Moreover, progerin mRNA levels correlated positively with CRP (Figure 3D) (*r* = 0.0751, *p* = 0.42). Using multivariate regression analysis progerin mRNA correlated independently with BMI.

## 3. Discussion

This study shows that mRNA levels of the aging related lamin A splice variant progerin, associated with premature aging in HGPS, were significantly upregulated in subjects with BMI ≥ 25 kg/m^2^. Moreover, our data revealed a significantly positive correlation of BMI with progerin mRNA. These data provide to our knowledge for the first-time evidence for a possible involvement of progerin in previously observed accelerated aging of overweight and obese individuals potentially limiting their longevity.

The occurrence of cardiovascular atherosclerotic diseases increases with age and the grade of obesity and further depends on risk factors such as hypertension, hyperglycemia, and hypercholesterolemia. Senescent endothelial cells are predominantly found at atherosclerotic sites in blood vessels, favoring the formation of atheromatous plaque [21]. Progerin, in turn is associated with cellular senescence due to shortened telomeres, DNA-damage, and metabolic and epigenetic alternations in HGPS. It induces endoplasmic reticulum stress in vascular smooth muscle cells and accelerates atherosclerosis [22]. Since progerin mRNA is also found in normal aged individuals, its contribution to physiologic aging processes seems reasonable.

With respect to our observation that premature aging related progerin mRNA highly correlated with BMI, a recently published meta-analysis of 87 observational studies revealed that BMI was negatively related to telomere length, another marker associated with premature aging [23]. Dietary restriction was already linked to longevity in 1917 by Osborne et al. [24]. A possible link between nutrient intake and activation of progerin expression through mammalian target of rapamycin (mTOR) signaling pathways was already discussed by Blagoskonny et al. [25]. Our own previously published data suggest that increased levels of progerin mRNA in the human heart were related to inverse cardiac remodeling with reduced ejection fraction which further might negatively impact longevity [26]. Moreover, we could show that enhanced progerin mRNA levels in diseased hearts with dilated cardiomyopathy were related to increased progerin protein levels in the nuclear compartment of cardiomyocytes co-expressing markers for apoptotic cell death. These observations suggest that increased mRNA levels might indeed translate to active progerin protein accumulation leading to deterioration of cardiac function.

In accordance with increased levels of the alternatively splicing product progerin, a recently published study supports the assumption that alternative splicing increases with aging in general and could lead to progression of disease [27].

Our results also showed that progerin mRNA was positively correlated with CRP. This might suggest an association of progerin with an inflammatory response triggering accelerated aging. Moreover, we found an increase of the acute phase protein CRP in patients with BMI ≥ 25, indicating a higher systemic inflammatory status in the overweight group. This is consistent with prior findings where obesity was considered to predispose to local and systemic inflammation with ongoing activation of immune cells [28].

Similar to obesity, an increase in chronic inflammation is observed in elderly, impairing innate and adaptive immune defense and contributing to an earlier onset of frailty [29]. Aging is associated with a decline in immune system function [30] and dysregulated innate and adaptive immune systems [31], resulting in a higher susceptibility for infections and chronic neurologic diseases [32].

Interestingly, it has been shown that levels of pro-inflammatory cytokines in old animals can be reduced to levels of young through caloric restriction [33]. Chronically elevated levels of pro-inflammatory markers, such as interleukin 6 (IL-6) or tumor necrosis factor-α (TNF-α), are key features of aging since low-grade inflammatory activity in the elderly is common. This pro-inflammatory environment has been defined as “inflammaging”. However, the mechanisms that cause and preserve high levels of these markers in aging processes are poorly understood [34,35].

Revechon et al. found that progerin expression in subcutaneous adipose tissue (sWAT) of rare progerin expressing mice is activating systemic inflammatory response. Furthermore, they found progerin expression in healthy human sWAT. Consecutively they speculated that mechanisms similar to the mouse model occur in human subcutaneous fat and that progerin accumulates during aging triggering a cascade of events as increased proliferation, senescence, cell death, and induction of systemic inflammation [36]. Inflammatory cytokines and shear stress lead to injury and apoptosis of endothelial, vascular smooth muscle cells, and pericytes in arterial walls. Those are renewed by reparative progenitor cells migrating into the damaged areas. Pacheco et al. observed that progerin interferes with critical progenitor cell functions impairing vascular repair. They further discussed progerin expression in the context of physiological aging causing atherosclerosis [37].

### Limitations

One limitation of our study is its reliance on height, weight, and preexisting conditions at a single point in time. Although BMI is not a perfect measure of adiposity, since it does not distinguish fat from lean body mass, height and weight are more easily measured than other indexes of excess adiposity, such as waist circumference [38]. Finally, an important limitation in terms of generalizability is the fact that the study population was restricted to patients of a cardiology outpatients’ unit. However, the population consisted of various cardiovascular diseases associated to conditions of limited longevity. This study is based on progerin mRNA levels determined by RT-PCR. The use of other methods investigating DNA, epigenetics, or protein levels may add further information in this field.

## 4. Materials and Methods

### 4.1. Study Population

For this cross-sectional retrospective analysis, we consecutively included Caucasian patients over 18 years of age of both sexes presenting at our cardiology outpatients’ unit. Patients were consecutively included between 2014 and 2016. Diagnoses are depicted in Table A1. Most of the patients suffered from DCM, followed by ICM, hypertrophic cardiomyopathy and other cardiovascular disease. There were no statistical differences in the distribution of disease entities between the groups.

BMI was calculated as weight in kilograms divided by height in meters squared and normal weight was defined as BMI < 25 kg/m^2^, whereas overweight was defined as BMI ≤ 25.0 kg/m^2^, according to the classification of World Health Organization (WHO) [39]. For measurement of progerin and lamin A concentrations, EDTA blood samples were obtained from 111 patient of which 46 were normal and 65 overweight.

Patients were treated according to prevailing CHF guidelines [40]. Patients with acute heart failure, prior heart transplantation or moderate to severe chronic kidney disease were excluded.

The study conformed to the principles outlined in the Declaration of Helsinki and was approved by the local ethics committee of Medical University of Innsbruck as “Interventionary studies involving animals or humans, and other studies require ethical approval must list the authority that provided approval and the corresponding ethical approval code AM4077 (approved on 28.10.2010)”. All patients gave written informed consent for participation in the study.

### 4.2. RT-PCR of mRNA

Fasting whole blood samples were drawn and immediately centrifuged for 10 min at 3600× *g*. The buffy coat containing lymphocytes, monocytes, and granulocytes was extracted, resuspended in Trizol (Invitrogen) and stored at −80 °C until analysis. After extracting total mRNA reverse transcription into cDNA (QuantiTect RT kit, Qiagen GmbH, 40724 Hilden, Germany) was performed according to manufacturer’s protocol.

For RT-PCR analysis of progerin, primers were designed and optimized spanning the splice junction site between exon-11 and 12 as published by us previously [26].

Exon spanning primers were verified on agarose gels. Amplification primers were:

Rpl32-F: 5′-AGTTCCTGGTCCACAACGTC-3′,

Rpl32-R: 5′-CTCTTTCCACGATGGCTTTG-3′

LMNA spec F: 5′-TCAGGAGCCCAGAGCCCCCAGAAC-3′

LMNA spec R: 5′-GGGTTATTTTTCTTTGGCTTCA-3′

The Cycling conditions for qPCR were 95 °C for 10 min (Activation), 95 °C for 15 s (Denaturation) and 60 °C for 1 min (Annealing and extension) up to 40 cycles. Using 2× SYBR green mastermix (Applied Biosystems, Waltham, MA 02451, USA) quantitative gene expression was calculated using the comparative ΔΔCt-method with RPL32 as a reference gene.

### 4.3. Statistical Analysis

Statistical analysis was performed using SSPS™ Statistics 24.0.0 (IBM, Armonk, NY, USA) and GraphPad Prism (Version 6.04, GraphPad Software, Inc, San Diego, CA 92108, USA) was used for generation of graphics. Quantitative variables are expressed as means ± standard deviation (SD) whereas categorical variables are presented as absolute values and percentages. For categorical variables fisher’s exact tests was used and independent *T*-test for to test for differences between the groups. Tests were two-tailed and a *p*-value of less than 0.05 was considered to indicate statistical significance. Correlation coefficients were determined by linear regression analysis (Spearman’s equation) and scatter plots were created.

## 5. Conclusions

We conclude that excessive weight and obesity are associated with increased levels of the aging related splice variant progerin and activation of systemic inflammation. Both are displaying putative mechanisms causing premature aging of overweight individuals limiting longevity.

## Figures and Tables

**Table 1:** Characteristics of Patients at Baseline.

Characteristic	Total Population *n* = 111		BMI < 25 (*n* = 46)	BMI ≥ 25 (*n* = 65)	*P* Value
**Age, years**		56 ± 15	54 ± 17	58 ± 13	0.1
Female sex, *n* (%)		32 (29)	18 (39)	14 (22)	0.14
**Body-mass-index, kg/m^2^**		**26.6 ± 4.8**	**22.4 ± 1.8**	**29.6 ± 4.0**	**0.000**
**NYHA class, *n* (%)**	***n* = 108**	**2.0 ± 0.7**	**1.8 ± 0.6**	**2.1 ± 0.7**	**0.015**
I		29 (27)	15 (33)	14 (22)	
II		55 (51)	25 (54)	30 (46)	
III		24 (22)	4 (9)	20 (31)	
IV		0 (0)	0 (0)	0 (0)	
Medical history, *n* (%)					
Smoking, *n* (%)	*n* = 108	49	17 (37)	32 (49)	0.17
Diabetes, *n* (%)		19 (17)	5 (11)	14 (22)	0.2
**Atrial fibrillation, *n* (%)**		**42 (38)**	**10 (22)**	**32 (49)**	**0.005**
LVEF, *n* (%)		38.2 ± 14.9	39.9 ± 14.2	37.0 ± 15.4	0.29
ACE-inhibitor, *n* (%)		55 (50)	24 (48)	31 (48)	0.7
ARB, *n* (%)		33 (30)	10 (22)	23 (36)	0.14
Beta-blocker		90 (81)	34 (74)	56 (86)	0.14
Clinical features					
NT-proBNP, ng/L	*n* = 108	2094 ± 4421	1748 ± 2118	2341 ± 5514	0.44
TroponinT, ng/L	*n* = 109	28 ± 53	32 ± 72	24 ± 33	0.48
Serum creatinine, mg/dL		1.2 ± 0.46	1.19 ± 0.54	1.21 ± 0.40	0.77
GFR, mL/min		54 ± 10	54 ± 10	54 ± 10	0.55
Urea, mg/dL		50 ± 37	48 ± 46	51 ± 30	0.73
**HbA1c, %**	*n* = 110	**5.9 ± 1.0**	**5.7 ± 1.1**	**6.1 ± 1.1**	**0.017**
**Triglycerides, mg/dL**		**135 ± 68**	**119 ± 59**	**145 ± 73**	**0.041**
Total cholesterol, mg/dL		172 ± 44	181 ± 43	166± 45	0.08
LDL, mg/dL	*n* = 109	107 ± 37	110 ± 38	105 ± 39	0.47
**HDL, mg/dL**	*n* = 109	**52 ± 19**	**59.0 ± 22.7**	**46.5 ± 14.0**	**0.003**
**Hs-CRP, mg/dL**	*n* = 110	**0.64 ± 1.07**	**0.28 ± 0.24**	**0.9 ± 1.33**	**0.01**
GGT, U/I		86 ± 146	87 ± 184	86 ± 114	0.96
GOT, U/I		29.2 ± 12.4	28.5 ±12.0	29.6 ± 12.7	0.65
Potassium		4.2 ± 0.5	4.3 ± 0.4	4.2 ± 0.5	0.12
Sodium		138 ± 13	139 ± 4	137 ± 17	0.19
Leucocyte count, G/l		7.7 ± 2.2	7.5 ± 1.9	7.9 ± 2.3	0.37
Thrombocytes, G/I		217 ± 71	223 ± 89	212 ± 54	0.44
Hemoglobin, G/I		140 ± 15	138	136	0.26

Plus–minus values are means ± standard deviation. *p* values refer to differences in continuous variables (Student’s *T*-test) or categorical variables (Fisher’s exact test). Percentages may not total 100 because of rounding. NYHA: New York Heart Association class, LVEF: left ventricular ejection fraction, ARB: angiotensin receptor blocker, GFR: Glomerular filtration rate, LDL: Low-density lipoprotein, HDL: High-density lipoprotein, Hs-CRP: high-sensitivity C-reactive protein, GGT: Gamma-glutamyltransferase, GOT: glutamic oxaloacetic transaminase.

## Abbreviations

BMI	Body-Mass-Index
CVS	Cardiovascular system
DCMP	Dilative cardiomyopathy
HGPS	Hutchinson–Gilford-Progeria syndrome
Hs-CRP	High-Sensitive C-Reactive Protein
HCMP	Hypertrophic cardiomyopathy
ICMP	Ischemic cardiomyopathy
NYHA	New York Heart Association
PCR	Polymerase Chain Reaction

## Appendix

**Table A1:** Diagnosis of included patients.

	DCMP	ICMP	HCMP	Others	Total
BMI < 25 kg/m^2^, *n* (%)	22 (49)	13 (28)	5 (11)	6 (13)	46
BMI ≥ 25 kg/m^2^, *n* (%)	37 (57)	18 (28)	7 (11)	3 (5)	65
Total	59	31	12	9	111

DCMP: Dilative cardiomyopathy, ICMP: Ischemic cardiomyopathy, HCMP: Hypertrophic cardiomyopathy; Pearson Chi-Square-test: *p* = 0.681.
